# Attitudes, practices, and determinants of community care-seeking behaviours for fever/malaria episodes in the context of the implementation of multiple first-line therapies for uncomplicated malaria in the health district of Kaya, Burkina Faso

**DOI:** 10.1186/s12936-022-04180-z

**Published:** 2022-05-30

**Authors:** Jean Moise Tanga Kaboré, Mohamadou Siribié, Denise Hien, Issiaka Soulama, Nouhoun Barry, Yacouba Nombré, Frederic Dianda, Adama Baguiya, Alfred Bewendtaoré Tiono, Christian Burri, André-Marie Tchouatieu, Sodiomon Bienvenu Sirima

**Affiliations:** 1 Groupe de Recherche Action en Santé (GRAS), P.O Box 10248, Ouagadougou 06, Burkina Faso; 2 Swiss Tropical and Public Health Institute, Basel, Switzerland; 3 University of Basel, Basel, Switzerland; 4 International Vaccine Institute (IVI), Seoul, Republic of Korea; 5 Programme National de Lutte contre le Paludisme, Ouagadougou, Burkina Faso; 6 Kaya Health and Demographic Surveillance System (Kaya-HDSS), Institut de Recherche en Sciences de la Santé (IRSS), Ouagadougou, Burkina Faso; 7 Medicines for Malaria Venture (MMV), Geneva, Switzerland

**Keywords:** Malaria, Care-seeking behaviour, Multiple first-line therapies, Burkina Faso

## Abstract

**Background:**

Malaria case management relies on World Health Organization (WHO)-recommended artemisinin-based combination therapy (ACT), and a continuous understanding of local community knowledge, attitudes, and practices may be a great support for the success of malaria disease control efforts. In this context, this study aimed to identify potential facilitators or barriers at the community level to inform a health district-wide implementation of multiple first-line therapies (MFT) as a new strategy for uncomplicated malaria case management.

**Methods:**

A community-based cross-sectional study using a mixed-method design was carried out from November 2018 to February 2019, in the health district (HD) of Kaya in Burkina Faso. Quantitative data were collected using a standardized questionnaire from 1394 individuals who had fever/malaria episodes four weeks prior to the survey. In addition, 23 focus group discussions (FGDs) were conducted targeting various segments of the community. Logistic regression models were used to assess the predictors of community care-seeking behaviours.

**Results:**

Overall, 98% (1366/1394) of study participants sought advice or treatment, and 66.5% did so within 24 h of fever onset. 76.4% of participants preferred to seek treatment from health centres as the first recourse to care, 5.8% were treated at home with remaining drug stock, and 2.3% preferred traditional healers. Artemether-lumefantrine (AL) was by far the most used anti-malarial drug (98.2%); reported adherence to the 3-day treatment regimen was 84.3%. Multivariate analysis identified less than 5 km distance travelled for care (AOR = 2.7; 95% CI 2.1–3.7) and education/schooling (AOR = 1.8; 95% CI 1.3–2.5) as determinants of prompt care-seeking for fever. Geographical proximity (AOR = 1.5, 95% CI 1.2–2.1), having a child under five (AOR = 4.6, 95% CI 3.2–6.7), being pregnant (AOR = 6.5, 95% CI 1.9–22.5), and living in an urban area (AOR = 2.8, 95% CI 1.8–4.2) were significant predictors for visiting health centres. The FGDs showed that participants had good knowledge about malaria symptoms, prevention tools, and effective treatment. Behaviour change regarding malaria treatment and free medication for children under five were the main reasons for participants to seek care at health centres.

**Conclusions:**

The study showed appropriate knowledge about malaria and positive community care-seeking behaviour at health centres for fever/malaria episodes. This could potentially facilitate the implementation of a MFT pilot programme in the district.

*ClinicalTrials.gov Identifier: *NCT04265573.

## Background

In 2019, 229 million new malaria cases and 409,000 deaths occurred worldwide, with 94% of cases and 94% of the deaths occurring in Africa [1]. The gains made this century against malaria have plateaued, and the disease continues to kill a child every 2 min. Malaria is responsible for an unacceptable number of hospital admissions and outpatient visits and greatly contributes to poverty in most sub-Saharan African regions [1].

Despite the progress made in malaria control, the disease remains the leading cause of morbidity and mortality in Burkina Faso, and is endemic, with a peak during the rainy season [2, 3]. According to the annual national health report 2018, malaria was responsible for 61.5% of hospitalizations and 30.5% of deaths in the country and an estimated 11 million uncomplicated malaria cases [2]. At the primary health facilities, 60.4% of admissions and 40% of deaths were associated with malaria, especially in children under five years of age [2].

To address the malaria burden in Burkina Faso, the national health authorities have implemented several programmes and malaria-control strategies at health facility and community levels. These include accurate malaria diagnosis and prompt treatment of uncomplicated cases; the introduction, in 2016, of a free healthcare policy for pregnant women and children under five years of age [4]; and a recommendation to use artemisinin-based combinations therapy (ACT) (5). This class of medicines was adopted and classified as first- and second-line treatments of malaria since2005, and updated in 2017. Community-based interventions are also being implemented, e.g., community case management of malaria since 2010 and the Seasonal Malaria Chemoprevention (SMC) strategy since 2014, targeting children aged between 3 and 59 months [3].

Current efforts to further control and eliminate malaria greatly depend on the perceptions, adherence, and acceptability of anti-malarial interventions. Early care-seeking behaviour associated with prompt diagnosis and treatment with ACT are key components to preventing a mild case of malaria from progressing into severe disease and death [3, 5–8]. Several studies across African countries have investigated community care-seeking practices and perceptions towards malaria as a disease [9–14]. These concluded that most frequently, seeking treatment is delayed; and poor access to health care providers or caregivers’ lack of education and awareness of malaria symptoms [1, 6] result in many febrile children not receiving adequate and prompt diagnosis and treatment. In addition, several studies have demonstrated that caregivers of children stop the malaria treatment with ACT prematurely upon resolution of initial symptoms [8, 15, 16]. Given that local beliefs around disease also influence attitudes and adherence to malaria treatment, the US President’s Malaria Initiative in collaboration with the NMCP, has agreed that one programme’s key performance indicator (KPI) is that 100% of the population knows two methods to prevent malaria, and another KPI is that 90% understand the need to seek treatment within 24 h of symptom onset [3].

With the development of artemisinin resistance in South-East Asia [17–20] and the risk of its spread to sub-Saharan Africa [21, 22], malaria control strategies have to be adjusted and adapted. Thus, optimising the use of current artemisinin-based combinations still efficacious in Burkina [23, 24] is crucial. To extend the therapeutic life of artemisinin-based combinations by reducing drug pressure and slowing down the spread of resistance [25, 26], the simultaneous use of multiple combinations as first-line therapy for malaria treatment has been suggested [25, 27]. The communities’ use of health services associated with early care-seeking plays an important role in the success and sustainability of any malaria control strategy [8, 28] including multiple first-line treatments.

Determinants of care-seeking behaviours, as well as practices and attitudes towards febrile episodes, were assessed among community members in the HD of Kaya in Burkina Faso to inform the implementation of the MFT pilot programme.

## Methods

### Study setting

**Fig. 1:**
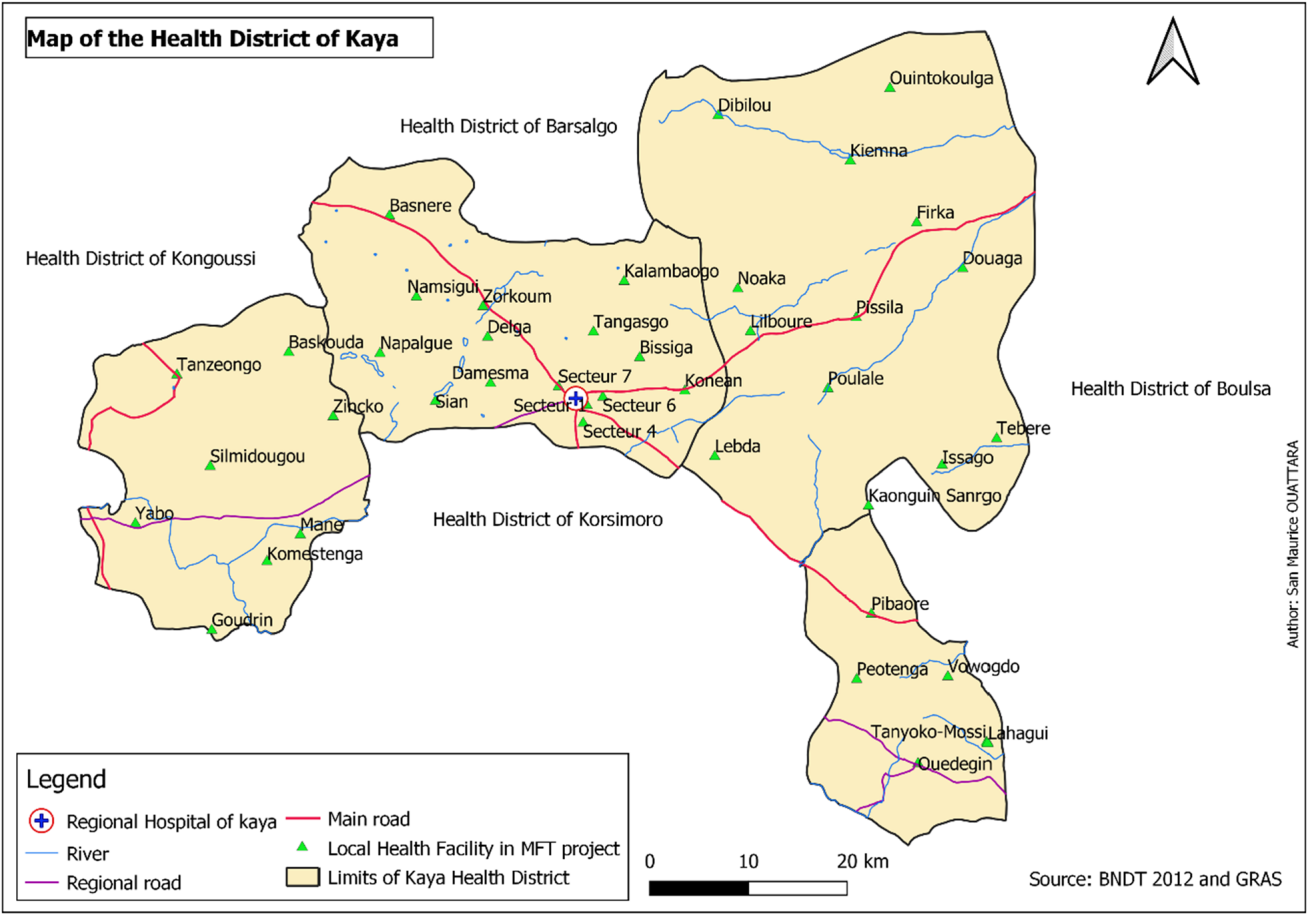
Map of Heath facilities in the HD of Kaya

This study was conducted ahead of the MFT pilot programme implementation, between November 2018 and February 2019. The study was conducted in the HD of Kaya (Fig. 1), located in the North-Central region, 100 km from Ouagadougou, the capital city of Burkina Faso. The HD of Kaya is composed of four communes, one urban (Kaya), and three rural (Mané, Piboaré, and Pissila) with a population of about 407,311 inhabitants in 2018. The district has 215 villages covering an area of 3617 km^2^, and 40 public health facilities, including 39 primary health care centres and one medical centre in 2018. There were four private and faith-based health facilities in Kaya. The regional hospital in Kaya is the region’s referral hospital. In the HD of Kaya, 191,771 uncomplicated malaria cases were recorded in 2018, with an incidence rate of 1.27 per person-year and 0.4 per person-year in children less than five years of age and the total population, respectively [2]. In the same year, 98.3% of the malaria cases were treated by ACT.

### Study design and populations

This was a community-based cross-sectional study with a mixed-method design.

#### Quantitative household survey

A quantitative household survey was carried out using a structured questionnaire during the high malaria transmission season in November 2018. The questionnaires were administered to all individuals, either directly or to their parents/caregivers, in households having had a fever within the 28 days preceding the survey. The subjects who had a fever in the household were categorized into four different groups: children under five, 5 to 15 years, 16 to 45 years, and pregnant women. Those subjects aged at least 18 years or emancipated minors (individuals aged below 18 years but married or with a child) were directly interviewed but if the subject having had fever was a minor (children and adolescents below 18 years), the questionnaire was administered to their parents/caregivers. The respondent was a person who could provide accurate information about the febrile episode and its management. Thus, a parent/caregiver was able to respond for more than one person in the household.

#### Qualitative survey

Focus group discussions (FGDs) were conducted with community members. The study populations included the head of household (male participants), mothers of children under five years of age, adult men, pregnant women, and community leaders. They were selected for their role in the care-seeking behaviours for suspected malaria episodes.

### Sample size and sampling strategy

For the quantitative survey, the study participants were selected through multi-stage sampling. In the first stage, ten public health centres were randomly selected from the list of health centres in the district. In the second stage, in each health facility, two villages or sectors were randomly selected to participate in the survey. In the last stage, households were randomly selected within the selected villages to take part in the survey using a “random walk” method. All individuals in a household having had a fever during the past four weeks were interviewed directly or interviews were done with their parents/caregivers.

The sample size calculation is described elsewhere [27]. Briefly, it was estimated at 408 with a confidence level of 95% and a desired power of 80%, from each of the three target groups of population (children under five, 5–15 years and 16–45 years) having had fever [27]. Pregnant women who have had fever were recruited during the survey at community level as long as the fieldworkers identified them. Assuming a rate of imponderables of 10%, the estimated sample size was 450 participants with fever in each age group.

In addition, FGDs were conducted with community members who are potential beneficiaries of care. Participants were randomly and purposively recruited for FGDs with the assistance of the heads of health centres. First, five specific groups in the community were identified to participate in the group discussions sessions: heads of household (male participants), mothers of children under 5, adult men, pregnant women, and community leaders. For each identified group, five health centres were randomly selected from the list of all health centres. Afterwards, with the assistance of the head of the health centre, participants were purposively identified and invited to take part in the discussion sessions. Sensitive to social and cultural issues, men and women were not mixed in the same FGD session.

### Variables’ definition

The outcome variables included (i) prompt care-seeking behaviour and (ii) care-seeking at health centres. The former was defined as seeking care within 24 h of fever onset. The latter comprised visiting primary health centres, medical centres, the regional hospital, and private clinic for fever within four weeks before the survey. Exploratory variables that could have a potential effect on populations’ care-seeking behaviours ranged from socio-demographic factors, accessibility to providers and the quality of services delivered by providers.

The socio-demographic factors of study participants included: age, gender, residence area, occupation, education status, and household size. The accessibility factors were defined as the distance travelled for care, cost accessibility and mode of transport to the health provider. The services factors delivered by providers were categorized as good reputation of provider, personal previous experience and availability of malaria medicines. The quality of the services was established based on either the good reputation, the previous positive experience in the same health facility and malaria medicines availability.

The management of febrile episodes at health centres was assessed through malaria rapid tests performed, treatment received and adherence to medication reported by the population.

### Recruitment and data collection: HD of Kaya

#### Quantitative survey

Participants were interviewed in 1,036 sample households of 19 randomly selected villages or sectors through face-to-face interviews using a structured questionnaire. Twelve fieldworkers (nine data collectors and three supervisors) were recruited and trained during 3 days on the study procedures. The questionnaire was pretested and administered over approximately 20–30 min in French and Mooré, the most spoken languages of study participants. The respondents interviewed were adults (age over 18 years) or emancipated minors (age less than 18 years and married or had a child). When the patient’s age was less than 18 years, the interview was conducted with parents/caregivers. A household was defined as a family unit where the head of the household, his spouse (s) and other relatives live together and share their income. Data collected were related to (i) socio-demographic status of participants, which could influence the care-seeking behaviours; (ii) care-seeking behaviours for febrile episode; (iii) factors influencing care-seeking behaviours; and (iv) access to effective management of fever/malaria, including treatment practices. Information on treatment practices was collected through self-reporting by study participants (malaria rapid test performed, treatment received and adherence to medication).

#### Qualitative survey

Data were collected through 23 FGDs conducted with community members to understand their knowledge on malaria disease (malaria transmission, symptoms, treatment and preventive methods), attitudes and practices towards fever/malaria and its management (Table 1). The FGD guide was developed by the study social scientists, pretested and administered to selected participants. Five social scientists with master’s degrees were recruited and trained for 3 days. They conducted the FGDs in the village where participants lived, at the residence of one participant or at the health centre. Each FGD included 7–10 participants and lasted for about 45 to 90 min. The discussions were audio-recorded, transcribed, and translated into French by the social scientists for analysis. The discussions were conducted in the local language (Mooré) using semi-structured interview guides grouped in two sections. The first section explored the community’s knowledge about malaria including its morbidity burden. The second section was dedicated to patterns of care-seeking behaviour.

**Table 1:** FGDs conducted with communities’ members

Group identified	Number of FGDs
Community key opinion leaders	5
Heads of household (male participants)	6
Mothers of children under 5	5
Pregnant women	4
Adult men	3
Total	23

### Data management and analysis

After data collection, quantitative data were entered by two data clerks using EpiData and analysed with Stata version 16 (StataCorp. 2016. Stata Statistical Software: Release 16. College Station, TX: StataCorp LP.). Univariate descriptive analyses such as frequencies, proportions, means or median were used to describe socio-demographic characteristics of the study populations, communities’ attitudes and practices regarding febrile episodes/malaria and access to effective malaria case management including diagnosis and treatment. Chi-square and Fisher’s exact tests were used to compare the proportions. Factors influencing care-seeking behaviours including time taken to seek treatment and visiting health centres were explored through bivariate and multiple logistic regression. The statistical significance was set at p < 0.05. Odds ratio and 95% confidence interval were used to measure the associations between the dependent and independent variables. A variable with a p-value of 0.20 or below in the tests of significance in the bivariate analyses was exported to the multivariate regression model. Likelihood Ratio Test was used to compare different models. The final model was obtained by backward elimination and was assessed for its fitness to the data by the Hosmer–Lemeshow goodness of fit test.

Data-derived codes developed through deductive and inductive coding and retrieving was used during analysis for qualitative data. Indeed, all transcripts were coded line-by-line using pre-set themes (deductive approach). Further codes were added for emerging themes (inductive approach). The qualitative data software programme NVivo (QSR International Pty Ltd. 2020) was used to organize all qualitative data and prepare these for analysis.

### Ethical considerations

Ethical approval was obtained from the National Health Ethics Committee of Burkina Faso (2017–6-075/ CERS). The study purpose and procedures were explained in the local languages to potential participants by study staff. Upon explaining the objectives of the study, written consent was obtained from all respondents and confidentiality was maintained. All procedures followed were carried out per the Helsinki Declaration version 2013, Fortaleza.

## Results

### Socio-demographic characteristics of study participants

The socio-demographic characteristics of the study participants are shown in Table 2. A total of 1394 subjects who had fever within 28 days before the survey were interviewed during the household survey of which 57.7% were females. One-third of subjects were less than five years of age (33.1%) and the proportion of pregnant women was 2.5% (34/1394). The mean age with standard deviation (SD) was 2.4 ± 1.2 years; 8.5 ± 3.0 years and 27.6 ± 8.7 years for children under five years, 5–15 years and participants 16 years and above, respectively. Three quarters (75.5%) of households visited had more than six persons living in the household. The majority of respondents, 52.2% (731/1394) were the mothers of subjects with fever and 11.6% (162/1394) were fathers. In total, 69% of respondents had never been to school whereas 8.6% reported having secondary or higher education levels. Over three quarters (77.7%) were farmers, followed by housewives (7.3%) and employees or merchants (6.3%).

**Table 2:** Socio-demographic characteristics of study participants

	Children < 5 years	5–15 years	16 years and above	Pregnant women	Total
	Frequency (%)	Frequency (%)	Frequency (%)	Frequency (%)	Frequency (%)
Target group of population with fever	462 (33.1)	460 (33.0)	438 (31.4)	34 (2.5)	1394 (100)
* Mean age (SD)*	*2.3 (1.2)*	*8.5 (3.0)*	*27.6 (8.7)*	*25.3 (6.4)*	*12.9 (12.0)*
Gender					
Male	240 (51.9)	213 (46.3)	137 (31.3)	0 (0.0)	590 (42.3)
Female	222 (48.1)	247 (53.7)	301 (68.7)	34 (100.0)	804 (57.7)
Residence area					
Rural	435 (94.2)	399 (86.7)	296 (67.6)	29 (85.3)	1159 (83.1)
Urban	27 (5.8)	61 (13.3)	142 (32.4)	5 (14.7)	235 (16.9)
Household size					
≤ 6	109 (23.6)	105 (22.8)	111 (25.3)	16 (47.1)	341 (24.5)
> 6	353 (76.4)	355 (77.2)	327 (74.7)	18 (52.9)	1,053 (75.5)
* Median (IQR)*	*9 (6)*	*10 (6)*	*9 (8)*	*7 (9)*	*9 (6)*
Relationship of respondents with patients					
Mother	357 (77.3)	306 (66.5)	68 (15.5)	0 (0.0)	731 (52.4)
Father	72 (15.6)	77 (16.7)	12 (2.7)	1 (2.9)	162 (11.6)
Self	0 (0.0)	4 (0.9)	246 (56.2)	30 (88.2)	280 (20.1)
Others	32 (6.9)	73 (15.9)	112 (25.6)	3 (8.8)	220 (15.8)
Missing data	1 (0.2)	0 (0.0)	0 (0.0)	0 (0.0)	1 (0.1)
Education level of respondents					
None	370 (80.1)	287 (62.4)	283 (64.6)	22 (64.7)	962 (69.0)
Informal education	35 (7.6)	42 (9.1)	30 (6.9)	4 (11.8)	111 (8.0)
Primary school	27 (5.8)	97 (21.1)	53 (12.1)	4 (11.8)	181 (13.0)
Secondary and higher	18 (3.9)	27 (5.9)	71 (16.2)	4 (11.8)	120 (8.6)
Missing data	12 (2.6)	7 (1.5)	1 (0.2)	0 (0.0)	20 (1.4)
Occupation of respondents					
Farmer	374 (81.1)	374 (81.3)	313 (71.5)	23 (67.7)	1083 (77.7)
Housewife	50 (10.8)	18 (3.9)	28 (6.4)	5 (14.7)	102 (7.3)
Employee/Merchant	25 (5.4)	29 (6.3)	42 (9.6)	3 (8.8)	99 (6.3)
Student	4 (0.9)	24 (5.2)	31 (7.1)	2 (5.9)	61 (4.4)
Others	7(1.5)	10 (2.1)	16 (3.7)	0 (0.0)	19 (2.1)
Missing data	2 (0.4)	5 (1.1)	8 (1.8)	1 (2.9)	6 (1.6)

SD: standard deviation; IQR: interquartile range

### Participants’ knowledge about malaria

Participants’ knowledge about malaria was explored through FGDs. Most of the respondents had good knowledge about malaria symptoms, prevention tools and effective treatment. They knew that fever is the main symptom of malaria, associated with other symptoms.

“When children have malaria, their bodies get hot (fever) and they vomit. Adults also get hot and vomit. Then, they say that he has “weogo” [“weogo” is the local name of malaria], that he has malaria. There are also headaches, vomiting” (FGD06, adult men, Pibaoré).

Participants agreed and recognized that malaria is transmitted by mosquitoes. They believed the cause of malaria had changed over time, as they said:

“In the past, it was said that it is food consumption that causes malaria, but today this is not quite true. It is only the mosquitoes and the unsanitary conditions that cause malaria” (FGD 07, Adult men, Napalgué).

“So, if mosquitoes can cause malaria, it is when they bite a person who has malaria and when they bite another person, they transmit malaria to that person” (FGD04, Mothers of children under five, Kaya).

Some main preventive measures against malaria are well identified and known by the local populations in the HD of Kaya; they stated:

“We sleep under bed nets, which protects us from malaria.” (FGD 07, Adult men, Napalgué)

"In the families, we use mosquito nets at night, we also clean the houses and manage the waste-water well so that mosquitoes cannot proliferate. Insalubrity is favourable for mosquitoes" (FGD11, Pregnant women, Ouedeguin).

### Care-seeking behaviours of participants towards malaria

As shown in Table 3, from 1394 individuals having experienced fever/malaria episodes in the past four weeks, only 28 patients did not take any care-seeking action while 98.0% (1366/1394) sought advice or treatment. Of those who acted, two-thirds (66.5%; 95% CI: [639–68.9%]) sought treatment within 24 h of fever onset; 76.8% (1043/1366) of patients chose health centres as the first choice of provider, followed by community health workers (CHWs) (9.5%), self-treatment at home with remaining drug stock (5.8%). Only 2.4% preferred to seek traditional healers for care.

**Table 3:** Malaria care-seeking behaviours per group

	Children < 5 years	5–15 years	16 years and above	Pregnant women	Total	p-value
	Frequency (%)	Frequency (%)	Frequency (%)	Frequency (%)	Frequency (%)	
N	462	460	438	34	1394	
Sought treatment						
Yes	454 (98.3)	456 (99.1)	424 (96.8)	32 (94.1)	1366 (98.0)	0.03
No	8 (1.7)	4 (0.9)	14 (3.2)	2 (5.9)	28 (2.0)	
Time of care-seeking						
Prompt treatment	311 (68.5)	307 (67.3)	266 (62.7)	20 (62.5)	904 (66.2)	0.01
Not prompt treatment	143 (31.5)	149 (32.7)	152 (35.9)	12 (37.5)	456 (33.4)	
Missing information	0 (0.0)	0 (0.0)	6 (1.4)	0 (0.0)	6 (0.4)	
First location of care-seeking provider						
Health Centres	387 (85.2)	340 (74.6)	287 (67.7)	29 (90.6)	1043 (76.4)	< 0.001
Community health workers	25 (5.5)	55 (12.1)	47 (11.1)	2 (6.3)	129 (9.4)	
Family stock	26 (5.7)	27 (5.9)	26 (6.1)	0 (0.0)	79 (5.8)	
Traditional healer	7 (1.4)	11 (2.4)	13 (3.1)	1 (3.1)	32 (2.3)	
Private pharmacy	2 (0.4)	9 (2.0)	29 (6.8)	0 (0.0)	40 (2.9)	
Street vendor	5 (1.1)	13 (2.8)	17 (4.0)	0 (0.0)	35 (2.6)	
Missing information	2 (0.4)	1 (0.2)	5 (1.2)	0 (0.0)	8 (0.6)	
Reasons for choosing a provider^a^						
Proximity	214 (47.4)	244 (53.6)	231 (55.0)	19 (59.4)	708 (52.1)	0.09
Availability of malaria drugs	155 (34.3)	143 (31.4)	123 (29.3)	3 (9.4)	424 (31.2)	0.02
Qualified health workers	138 (30.5)	131 (28.8)	136 (32.4)	11 (34.4)	416 (30.6)	0.67
Good reputation of providers	50 (11.1)	90 (19.8)	75 (17.9)	4 (12.5)	219 (16.1)	0.003
Personal good experience	59 (13.1)	54 (11.9)	45 (10.7)	2 (6.3)	160 (11.8)	0.55
Low cost of care	52 (11.5)	19 (4.2)	20 (4.8)	0 (0.0)	91 (6.7)	< 0.001
Other reasons	35 (7.7)	23 (5.1)	32 (7.6)	5 (15.6)	95 (70)	0.07
Distance travelled for care^b^ (km)						
< 5	306 (71.8)	362 (84.6)	304 (77.4)	17 (53.1)	989 (77.3)	< 0.001
≥ 5	107 (25.1)	63 (14.7)	75 (19.1)	13 (40.6)	258 (20.2)	
Missing information	13 (3.1)	3 (0.7)	14 (3.6)	2 (6.3)	32 (2.50)	
Means to reach health care provider^b^						
Walking	119 (27.9)	95 (22.2)	75 (19.1)	5 (15.6)	294 (23.0)	0.001
Bicycle	192 (45.1)	144 (33.6)	82 (20.9)	10 (31.3)	428 (33.5)	
Motorbike	106 (25.0)	180 (42.1)	221 (56.2)	16 (50.0)	523 (40.9)	
Others	1 (0.2)	3 (0.7)	1 (0.3)	0 (0.0)	5 (0.4)	
Missing information	8 (1.9)	6	14 (3.69)	1 (3.1)	29 (2.3)	

^a^Multiple responses (percentages add to more than 100% as some patients had more than one reason of choosing a provider) ^b^we excluded those who have been treated at home with the family stock

These findings were further complemented by qualitative data from the FGDs and observational field notes, and identified the patterns of care-seeking with different providers. The concept of malaria disease has changed, and this mental change led to good care-seeking attitudes when community members have a febrile episode.

"As soon as you feel that something is wrong [sick], you quickly go to the health centre” (FGD03, Mothers of children under five, Sian)

“When a person has malaria, we go to the hospital where the doctor takes the blood and does testing, if it is malaria, he injects you (drug) and prescribes medication” (FGD20, Head of household, Kalambaogo).

"As the children have become fragile, even if they are given traditional medicines, they refuse. Therefore, you have to come here. So you have to come here [health centres], otherwise it won't heal” (FGD05, Mothers of children under five, Tanzéogo).

Health centres are by far the most-preferred place to seek treatment but some participants explained that traditional healers and remaining familial drug stock are also used.

“So far, we do still use decoctions (traditional healers), in addition to medicines and injections, because this allows us to boost the modern medicine in the treatment of the patient. We use acacia, mango, eucalyptus, guava leaves for the decoction" (FGD07, adult men, Napalgué).

“In my case, when it happens [fever], I first look for paracetamol. If it doesn't work, I come to the health centre” (FGD09, household head, Kaya)

“In families, there are several categories of malaria, so if someone goes to the dispensary once and is prescribed medication and gets well, after the cure, if another child is sick, they take the remaining medicine again to give to this child who is sick” (FGD12, Pregnant women).

### Malaria treatment practices reported by populations

The Table 4 shows the malaria treatment practices reported by the population. Almost three quarters (71.2%) of patients with fever were tested for malaria using a rapid diagnostic test (RDT). Of those tested, 96.7% were positive for malaria. For treatment received by patients having had a fever, three quarters (74.8%) received ACT as an anti-malarial drug. Almost all of malaria patients were treated with AL (98.2%) and the adherence level to a treatment regimen with ACT was 84.3%.

**Table 4:** Malaria treatment practices reported by populations

	Children < 5 years	5–15 years	16 years and above	Pregnant women	Total	p-value
	Frequency (%)	Frequency (%)	Frequency (%)	Frequency (%)	Frequency (%)	
N	462	460	438	34	1394	
Malaria RDT test performed						
Yes	348 (75.3)	334 (72.6)	285 (65.1)	24 (73.5)	992 (71.2)	< 0.001
No	108 (23.4)	126 (27.4)	153 (34.9)	9 (26.5)	396 (28.4)	
Unknown	6 (1.3)	0 (0.0)	0 (0.0)	0 (0.0)	6 (0.4)	
Malaria test result						
Positive	326 (93.7)	330 (98.8)	280 (98.2)	22 (88.0)	958 (96.6)	< 0.001
Negative	4 (1.1)	0 (0.0)	3 (1.0)	1 (4.0)	8 (0.8)	
Unknown	16 (4.6)	4 (1.2)	1 (0.4)	2 (8.0)	23 (2.3)	
Missing data	2 (0.6)	0 (0.0)	1 (0.4)	0	3 (0.3)	
Treatment received^a^						
Malaria drug						
ACT	319 (71.8)	350 (78.7)	310(75.8)	15(48.4)	994 (74.8)	< 0.001
Non-ACT drug^b^	11 (2.5)	11 (2.5)	21 (5.1)	5 (16.1)	51 (03.8)	< 0.001
Artemisinin monotherapy^c^	15 (03.4)	6 (1.4)	7 (1.7)	5 (16.1)	33 (02.5)	< 0.001
Non-Malaria drug						
Antipyretics	385 (86.7)	410 (92.1)	375 (91.7)	25 (80.7)	1195 (89.9)	0.01
Antibiotics	60 (13.5)	30 (6.7)	20 (4.9)	4 (12.9)	114 (23.5)	< 0.001
Unknown	17 (03.8)	8 (1.8)	15 (3.7)	2 (6.5)	42 (3.2)	0.31
Name of ACT received						
Artemether-lumefantrine	313 (98.1)	343 (98.3)	304 (98.1)	15 (100.0)	975 (98.2)	0.95
Other ACTs^d^	6 (1.9)	6 (1.7)	6 (1.9)	0 (0.0)	18 (1.8)	
Adherence of duration of treatment with ACT						
1–2 days	25 (7.8)	13 (3.7)	6 (1.9)	1 (6.7)	45 (4.5)	0.01
3 days	244 (76.5)	295 (84.5)	279 (90.9)	12 (80.0)	830 (83.6)	
> 3 days	47 (14.7)	39 (11.2)	22 (7.1)	2 (13.3)	110 (11.1)	
Missing information	3 (0.9)	2 (0.6)	3 (1.0)	0	8 (0.8)	

^a^Multiple responses (percentages add to more than 100% as some patients had more than one reason of choosing a provider)

^b^Quinine, sulfadoxine-pyrimethamine

^c^Artesunate and artemether injectable

^d^Pyronaridine-artesunate or amodiaquine-artesunate or dihydroartemisinin-piperaquine

### Factors associated with prompt care-seeking

Table 5 shows the factors associated with prompt treatment-seeking using bivariate and multivariate analysis. In the bivariate analysis, distance travelled for care less than 5 km (OR = 2.7; 95% CI 2.0–3.6), household size ≤ 6 people (OR = 1.4; 95% CI 1.1–1.9), schooling (OR = 1.8; 95% CI 1.4–2.5) were significantly associated with prompt treatment-seeking behaviours.

**Table 5:** Factors associated with prompt care-seeking

Covariates	Prompt treatment		Bivariate analysis		Multivariate Analysis	
	Yes Number (%)	No Number (%)	OR [95% CI]	p-value	AOR (95% CI)	p-value
Gender						
Male	384 (66.6)	193 (33.4)	1			
Female	520 (66.4)	263 (33.6)	1.0 [0.8–1.3]	0.96		
Target group of population						
< 5 years	311 (68.5)	143 (31.5)	1		1	
5–15 years	307 (67.3)	149 (32.7)	0.9 [0.7–1.3]	0.70	0.8 [0.6–1.1]	0.14
≥ 16 years	266 (63.6)	152 (36.4)	0.8 [0.6–1.1]	0.13	0.7 [0.5–0.9]	**0.02**
Pregnant women	20 (62.5)	12 (37.5)	0.8 [0.4–1.6]	0.48	1.0 [0.5–2.3]	0.90
Location of residence						
Rural	739 (65.6)	388 (34.4)	0.8 [0.6–1.1]	0.12		
Urban	165 (70.8)	68 (29.2)	1			
Household size						
≤ 6	237 (72.3)	91 (27.7)	1.4 [1.1–1.9]	**0.01**		
> 6	667 (64.6)	365 (35.4)	1			
Distance travelled for care						
≤ 5 km	699 (69.6)	306 (30.4)	2.7 [2.0–3.6]	** < 0.001**	2.8 [2.1–3.7]	** < 0.001**
> 5 km	117 (76.1)	138 (54.1)	1		1	
Education						
No schooling	660 (63.3)	383 (36.7)	1		1	
Schooling	226 (72.8)	71 (23.9)	1.8 [1.4–2.5]	** < 0.001**	1.8 [1.3–2.5]	** < 0.001**
Occupation						
Farmers/housewife	760 (65.9)	393 (34.1)	1			
Non farmers/housewife	132 (69.1)	59 (30.9)	1.2 [0.8–1.6]	0.39		

In bold significant p-value < 0.05; OR: Odds Ratio; AOR: Adjusted Odds Ratio; 95% CI 95% confidence interval

For the multivariate analysis, independent variables such as target group (age group), education level and distance travelled to care were included

From the multiple logistic regression analysis, distance from the provider (OR = 2.7; 95% CI 2.1–3.7), schooling (OR = 1.8; 95% CI 1.3–2.5) were found to be significantly associated with prompt treatment. People living < 5 km from the provider were 2.2 times more likely to seek treatment within 24 h of fever onset.

### Factors influencing care-seeking at health centres

Bivariate and multivariate logistic regressions were performed to assess independent factors associated with the attendance at health-centre providers as shown in Table 6. From those who sought treatment at health centres, geographical proximity to health centres, having children under 5 or being pregnant women, smaller household size and residence in an urban area significantly affect care-seeking behaviours after adjusting on education level. Most of the participants went to health centres because of proximity (AOR = 1.5, 95% CI 1.2–2.10; p = 0.01). In addition, having a child under five (AOR = 4.6, 95% CI 3.2–6.7), being a pregnant woman (AOR = 6.5, 95% CI 1.9–22.5), living in an urban area (AOR = 2.8, 95% CI 1.8–4.2), household size less than 6 members (AOR = 0.7, 95% CI 0.5–0.9) were found to be statistically associated with visiting health centres for febrile episodes. The bivariate analysis showed that the geographical proximity was not initially significantly associated with seeking treatment at a health facility (p = 0.62).

**Table 6:** Logistic regression analysis to identify independent significant variables associated with care-seeking at health centres

Covariates	Health centres as the first care provider		Bivariate Analysis		Multivariate analysis	
	Yes Number (%)	No Number (%)	Crude OR (95% CI)	p-value	AOR (95% CI)	p-value
Proximity						
Yes	546 (77.6)	158 (22.4)	1.1 [0.8–1.4]	0.62	1.5 [1.2–2.1]	**0.01**
No	496 (76.4)	153 (23.6)				
Good reputation of the provider						
Yes	215 (98.2)	4 (0.2)	19.9 [7.3–54.1]	** < 0.001**	33.6 [12.5–95.3]	** < 0.001**
No	827 (72.9)	307 (27.1)				
Low cost of care						
Yes	56 (61.5)	35 (38.5)	0.4 [0.3–0.7]	** < 0.001**	0.5 [0.3–0.8]	**0.002**
No	986 (78.1)	276 (21.9)				
Good personal experience						
Yes	113 (70.3)	47 (29.4)	0.7 [0.5–1.0]	**0.04**		
No	929 (77.9)	264 (22.1)				
Availability of malaria medicines						
Yes	342 (80.9)	81 (19.1)	1.4 [1.0–1.8]	**0.02**		
No	700 (75.3)	230 (24.7)				
Target group of population						
< 5 years	387 (85.6)	65 (14.4)	2.7 [2.0–3.8]	** < 0.001**	4.6 [3.2–6.7]	** < 0.001**
5–15 years	340 (74.7)	115 (25.3)	1.4 [1.1–1.8]	**0.04**	1.6 [1.2–2.3]	**0.002**
Pregnant women	29 (90.6)	3 (9.4)	4.4 [1.3–14.9]	**0.01**	6.5 [1.9–22.5]	**0.003**
16–45 years	287 (68.5)	132 (31.5)	1			
Gender						
Female	595 (76.3)	185 (23.7)	0.9 [0.7–1.2]	0.60		
Male	448 (77.5)	130 (22.5)	1			
Household size						
≤ 6	236 (72.4)	90 (27.6)	0.7 [0.6–1.0]	**0.03**	0.7 [0.5–0.9]	**0.04**
> 6	807 (78.2)	225 (21.8)	1			
Residence area						
Urban	186 (81.2)	43 (18.8)	1.4 [1.0–2.0]	0.08	2.8 [1.8–4.2]	** < 0.001**
Rural	857 (75.9)	272 (24.1)	1			
Education						
No schooling	805 (77.2)	238 (22.8)	1			
Any schooling	218 (73.9)	77 (26.1)	0.8 [0.6–1.1]	0.24	0.8 [0.6–1.2]	0.33
Occupation						
Farmer/housewife	892 (77.3)	262 (22.7	1			
Non Farmer/housewife	139 (73.9)	49 (26.2)	0.8 [0.6–1.2]	0.31		

In bold significant p-value < 0.05; OR: Odds Ratio; AOR: Adjusted Odds Ratio; 95% CI 95% confidence interval

For the multivariate analysis, factors associated with care-seeking behaviours at health centres were adjusted on education level

During the FGD, the main reasons for participants to seek care at health centres were described as: Geographical proximity, belief that malaria is a hospital-treated disease and availability of free medication for children under 5.

“Whether it is in the morning, or at night since the health centre is nearby, you don't even hang around” (FGD03, mothers of children under five, Damesma)

“You will not see any more patients in the health centres here if there is no more free health care. We see many children here because of the free health care” (FGD06, adult men, Piboaré).

“Before, people were not aware of the seriousness of the disease. Now, people are aware of it. They run directly with the patient to the hospital. In the case of malaria, people run quickly to see a health worker and the result is that they immediately recover their health (FGD14, community leaders, Mané).

## Discussion

This study was initiated before the implementation of the MFT pilot programme as a new malaria treatment strategy in the HD of Kaya to assess communities’ attitudes, practices and determinants of care-seeking behaviours for fever/suspected malaria episode. Overall, populations in the HD of Kaya have a good attitude regarding care-seeking behaviour for a febrile episode or suspected malaria. They sought advice or treatment for the majority of febrile episodes, most of the time within 24 h of fever onset and at health centres.

The proportions of populations seeking care for fever, who promptly sought care and got treated at health centres were 98%, 66.5% and 76.8%, respectively. Health centres were by far the first choice of care providers followed by CHWs, self-treatment using the anti-malarial drugs at home and traditional healers. Proximity to health centres, good reputation of providers, having a child under five, being pregnant or residing in an urban area were significantly associated with care-seeking at health centres while geographical proximity and education (schooling) were identified as determinants of prompt care-seeking within 24 h of fever onset.

In this study, communities sought advice or treatment for almost all febrile episodes (98%). Similar findings in care-seeking for fever were previously reported [29] and the results are consistent with the rate of malaria care-seeking reported by studies conducted in Liberia (98.5%) [30], Ethiopia (87.8%) [31], six African countries: Benin, Democratic Republic of Congo, Madagascar, Nigeria, Uganda and Zambia (82.6–95.5%) [32] and Laos (92%) [33]. The higher appropriate care-seeking behaviour rate in this study resulted partly from different efforts deployed in malaria control activities, including community sensitization based on CHWs activities, and social and behaviour change communication (school-based information campaigns, dramas). These activities resulted in improving people’s knowledge of malaria disease, which increased their willingness to take action after the onset of presumed malaria symptoms, especially fever.

Two-thirds of the study population sought treatment within 24 h of fever onset. Proximity to the care provider (< 5 km) was the main factor explaining this behaviour and practices. The findings of this study are consistent with results reported in the previous studies elsewhere [34–36]. However, the proportion of people promptly seeking care in this study is higher than results found in studies conducted in Equatorial Guinea (46.7%) [37] and Ethiopia (52.4%) [38]. In those studies settings, accessibility of providers was an issue and could have explained the lower proportion of prompt care-seeking. People living far from the provider are more likely to delay care-seeking. Proximity to a health provider meant better accessibility to the provider, which influenced care-seeking within the 24 h of fever onset. In line with Burkina Faso NMCP’s strategic plan that set the goal for at least 90% of the population knowing the advantages of receiving malaria treatment in the first 24 h of illness (5), this care-seeking behaviour is encouraging, as it will prevent mild cases from becoming severe, and hence reduce malaria-related mortality. The level of prompt treatment- seeking in this study could also be explained by the communities’ good knowledge about malaria reported during the FGDs. In addition, several studies showed that prompt treatment-seeking within 24 h of fever onset is strongly linked to the knowledge of malaria [31, 38, 39].

To manage febrile episodes, people in the community choose providers differently. In this study, health centres were the preferable providers for those seeking care for fever. The findings revealed that about three-quarters of study participants used health-centre services as the first point of call for treatment. Similar results were reported elsewhere [31, 33, 40]. People recognize that malaria is fatal if appropriate treatment is not given on time. To explore factors influencing the use of health-centre services multivariate logistic regression was performed. Proximity to health centres, having children under five, being pregnant, or residing in in urban areas were significantly associated with care-seeking at health centres.

Geographical proximity is a well-established, strong predictor of attending health centres and was widely demonstrated in many studies [33, 41–43]. This study showed the same result. People living far from health centres are less likely to seek treatment at those facilities. In this study, the mean distance from participants’ residences to health centres was 3.5 km. This reflects the availability of health centres in the study area, and might explain the higher number of people attending health centres for advice or treatment.

The behaviour preference to seek treatment at health centres is also associated with the location of residence. In this study, populations living in urban areas are more likely to seek treatment at health centres for fever than those in rural areas. Similar findings were reported in other studies conducted in sub-Saharan African countries [40, 44]. These findings suggest that people living in urban areas are more likely to have higher levels of education/schooling, which could increase their knowledge about malaria and have an impact on their willingness to use the health-centre services.

Parents having children under five, and pregnant women, are more likely to seek treatment at health centres compared to other age groups. The recent lifting of treatment fees for children under 5 and pregnant women and the sensitization of mothers of children through SMC delivery in Burkina Faso may explain our findings. The results are consistent with studies reporting that abolition of user fees for children under five increases the probability that caregivers would take them to health centres in case of febrile illness [6, 45, 46]. Indeed, during the FGDs, the access to free care for children was one of the main reasons given by caregivers for visiting health centres and was associated with an increase in the number of consultations at health centres.

Artemether-lumefantrine (AL) was used in the majority of all treatments with ACT given to febrile patients. This finding is in line with the NMCP guideline for malaria case management at public health centres and the community level in Burkina Faso [47]. AL is the most recommended first-line therapy for malaria, and the most available drug at public health centres and with CHWs. In addition, this finding revealed the higher pressure on AL by using it to treat all patients with malaria. Overall adherence to the ACT regimen was 84.5%. The adherence level is consistent with other studies conducted in Burkina Faso [48], Tanzania [49], Kenya [50]. This level of adherence is acceptable and could be attributed to the effect of the efforts of the malaria control programme. Nevertheless, 15.5% of patients were not adherent to the full three-day regimens of AL. The consequences of inappropriate, low-dose use of ACT, especially non-adherence to a treatment regimen associated with the permanent use of the same drug for malaria treatment, can lead to selecting artemisinin drug resistance [51].

The findings reported in this study should be considered along the following limitations and strengths. This study used a mixed-method design to collect data, which can be considered a strength. The qualitative part complemented the quantitative findings–during the FGD, populations described in their own words their care-seeking behaviour. The summary of findings was important to inform the implementation of MFT pilot programme.

One of the limitations in this study is the 4-week recall period used to collect information about the febrile episode that can lead to recall bias. In addition, data collected and presented here are self-reported, which might be associated with social desirability bias. The cross-sectional design of our study did not allow us to explore a causal relationship.

## Conclusions

This study conducted in the HD of Kaya showed that a large majority of the population sought treatment at health centres within the 24 h of fever onset. In addition, adherence to the ACT regimen was high and almost all patients were treated with the same artemisinin-based combination, AL. These results, combined with the good knowledge of malaria disease, could potentially facilitate a successful implementation of the MFT pilot programme, which is based on activities at health-centre level. The MFT programme could have significant benefits in fighting drug resistance by slowing the fixation of resistant strains and retarding selection pressure to the partner drugs used in artemisinin combinations. In tandem, continuous effort will be needed to sustain the population’s awareness of effective malaria case management and their prompt treatment-seeking behaviour upon onset of suspected malaria symptoms.

## Data Availability

The datasets generated and/or analysed during the current study are available from the corresponding author on reasonable request.

## Abbreviations

ACT: Artemisinin-based combination therapy
AL: Artemether-lumefantrine
CHWs: Community health workers
FGD: Focus group discussion
HD: Health district
KPI: Key performance indicator
MFT: Multiple First-line Therapies
NMCP: National Malaria Control Programme
RDT: Rapid diagnostic test
WHO: World Health Organization
